# Childhood TB epidemiology and treatment outcomes in Thailand: a TB active surveillance network, 2004 to 2006

**DOI:** 10.1186/1471-2334-8-94

**Published:** 2008-07-18

**Authors:** Rangsima Lolekha, Amornrat Anuwatnonthakate, Sriprapa Nateniyom, Surin Sumnapun, Norio Yamada, Wanpen Wattanaamornkiat, Wanchai Sattayawuthipong, Pricha Charusuntonsri, Natpatou Sanguanwongse, Charles D Wells, Jay K Varma

**Affiliations:** 1 Global AIDS Program, Thailand MOPH – U.S. CDC Collaboration, Nonthaburi, Thailand; 2 TB Program, Thailand MOPH – U.S. CDC Collaboration, Nonthaburi, Thailand; 3 Bureau of TB, Thailand Ministry of Public Health, Nonthaburi, Thailand; 4 Chiang Rai Provincial Public Health Office, Chiang Rai, Thailand; 5 Research Institute of Tuberculosis (Japan), Chiang Rai, Thailand; 6 AIDS, TB, STI and Leprosy Section, Office of Disease Prevention and Control 7, Ubon-ratchathani, Thailand; 7 Phuket Provincial Public Health Office, Phuket, Thailand; 8 Health Center 41, Bangkok Metropolitan Health Administration, Bangkok, Thailand; 9 Medicine Department, Bamrasnaradura Institute, Nonthaburi, Thailand; 10 Division of TB Elimination, U.S. Centers for Disease Control and Prevention, Atlanta, USA

## Abstract

**Background:**

Of the 9.2 million new TB cases occurring each year, about 10% are in children. Because childhood TB is usually non-infectious and non-fatal, national programs do not prioritize childhood TB diagnosis and treatment. We reviewed data from a demonstration project to learn more about the epidemiology of childhood TB in Thailand.

**Methods:**

In four Thai provinces and one national hospital, we contacted healthcare facilities monthly to record data about persons diagnosed with TB, assist with patient care, provide HIV counseling and testing, and obtain sputum for culture and susceptibility testing. We analyzed clinical and treatment outcome data for patients age < 15 years old registered in 2005 and 2006.

**Results:**

Only 279 (2%) of 14,487 total cases occurred in children. The median age of children was 8 years (range: 4 months, 14 years). Of 197 children with pulmonary TB, 63 (32%) were bacteriologically-confirmed: 56 (28%) were smear-positive and 7 (4%) were smear-negative, but culture-positive. One was diagnosed with multi-drug resistant TB. HIV infection was documented in 75 (27%). Thirteen (17%) of 75 HIV-infected children died during TB treatment compared with 4 (2%) of 204 not known to be HIV-infected (p < 0.01).

**Conclusion:**

Childhood TB is infrequently diagnosed in Thailand. Understanding whether this is due to absence of disease or diagnostic effort requires further research. HIV contributes substantially to the childhood TB burden in Thailand and is associated with high mortality.

## Background

TB causes 9.2 million illnesses and 1.7 million deaths annually [1]. About 10% of these TB cases occur in children, i.e., persons less than 15 years old [2-4]. In most countries, national TB programs (NTPs) focus case finding and treatment on pulmonary, acid-fast bacilli (AFB) smear-positive cases, which can be rapidly and reliably diagnosed and contribute greatest to community TB transmission [5]. Because most childhood TB cases are not smear-positive, children are given lower priority in NTPs. Historically, TB surveillance systems in high-burden countries only recorded age categories for smear-positive cases; as a result, few NTPs have been able to report reliably the total number of childhood TB cases diagnosed and treated annually [3]. The World Health Organization (WHO) recommends TB disease screening in children who live in the household of a smear-positive case, but few NTPs perform this routinely [6].

Despite the lack of attention paid by NTPs, childhood TB is an important public health problem. Because most children have primary, rather than reactivation, disease, childhood TB is considered a "sentinel" event, indicating recent TB transmission, often from an infectious, undiagnosed TB case in the same household [7]. Identifying and treating TB infection and disease in children can also provide long-term benefits to TB control, preventing future cases due to reactivation [7]. The global HIV pandemic has accelerated the urgency of TB control in children. Rates of childhood TB appear to be rising, particularly in countries with generalized HIV epidemics [3]. Multiple studies from sub-Saharan Africa have documented a markedly increased mortality rate for HIV-infected children with TB compared to HIV-uninfected children with TB or to HIV-infected children without TB [8-12].

WHO has recently called for more studies to define the global epidemiology of childhood TB, because the literature remains scant, dominated primarily by studies from industrialized countries and South Africa [13]. Few epidemiologic studies of pediatric TB have been published from Asia. Thailand is among the 22 high-burden TB countries on WHO's list and has a generalized HIV epidemic [1]. Notification rates for childhood TB in Thailand are markedly lower than in other countries; in fact, the proportion of all reported TB cases that occur in children (2.7%) is the lowest of the 22 high-burden countries [3]. We analyzed data from a multi-province demonstration project to understand characteristics, treatment outcomes, and risk factors for death and default in childhood TB cases.

## Methods

### Setting

The Thailand TB Active Surveillance Network is a demonstration project, implemented by the Thailand Ministry of Public Health (MOPH), Bangkok Metropolitan Administration (BMA), U.S. Centers for Disease Control and Prevention (CDC), and Research Institute of Tuberculosis, Japan (RIT), involving all districts in three provinces (Chiang Rai, Phuket, Ubon-ratchathani), selected districts in Bangkok (2 in 2005; 9 in 2006), and one national infectious diseases hospital (Bamrasnaradura Institute). In 2006, the catchment area included 59 public and 26 private healthcare facilities. In 2005, the last year for which published population estimates were available, the estimated number of persons aged 0 – 14 years old in the catchment area was 743,574, including 218,012 aged 0–4 years and 525,562 aged 5–14 years; for this calculation, we excluded the national infectious diseases hospital because this referral facility has no definable catchment area (unpublished data, Thailand National Statistics Department and BMA Office). A detailed description of this project, including methods, has been published elsewhere [14].

### TB diagnosis

Thai national guidelines recommend that childhood pulmonary TB (PTB) be diagnosed using a combination of factors, including: history of contact with a PTB patient known to be sputum smear-positive for acid-fast bacilli (AFB); suspected TB symptoms and signs; an abnormal chest radiograph, including pulmonary infiltrates and/or hilar or mediastinal lymphadenopathy; sputum or gastric aspirates that are smear-positive; and tuberculin skin testing. Because this project relied on data collected as part of routine TB control and most cases occurred in adults, limited information was collected about diagnostic tests other than chest radiography, sputum microscopy, and sputum culture. We did not collect data about tuberculin skin testing. Gastric aspirate specimens were classified as sputum specimens, in accordance with the routine TB program practice of classifying patients with abnormal chest radiographs and AFB-positive gastric aspirates as having smear-positive, pulmonary TB. In Thailand, criteria for EPTB diagnosis are less clearly delineated.

All public hospitals are able to process sputum for AFB smear microscopy. As part of this demonstration project, physicians were encouraged to send sputum specimens for mycobacterial culture at a province-level facility. All specimens were initially cultured on solid media, either Lowenstein-Jensen (LJ) or Ogawa medium, and, during mid-2005, additionally on liquid media with an automated reading instrument (Mycobacterial Growth Indicator Tube [MGIT]; BACTEC 960, Becton-Dickinson Corporation). Isolates underwent drug-susceptibility testing (DST) against first-line drugs (streptomycin, isoniazid, rifampin, pyrazinamide, ethambutol) at the Bangkok municipal or national TB reference laboratories.

A case of childhood TB was defined as a person aged 0 – 14 years old who was diagnosed with TB and treated for TB disease. A "new" case of TB was considered TB disease in a person who reported no or less than 1 month of previous TB treatment.

### TB treatment

Thai national guidelines recommend standardized, internationally-accepted treatment regimens for children. Treatment outcomes were defined using standard WHO categories [4].

### HIV diagnosis and treatment

In this demonstration project, nurses and physicians from public and private healthcare facilities were trained in HIV counseling and testing. They were encouraged to provide HIV counseling and testing to TB patients and referrals to HIV-related care and treatment to HIV-infected TB patients. No financial incentives were provided to patients or healthcare workers for HIV testing. Individual physicians used their own judgment about whether to measure CD4^+ ^T-cell lymphocyte counts (CD4), provide opportunistic infection (OI) prophylaxis or anti-retroviral therapy (ART), and manage other clinical conditions. When performed, blood for CD4 counts was usually drawn during the first month of TB treatment.

### Data collection and analysis

Data were collected on standardized forms that include questions about demographics, clinical history, laboratory and radiographic testing, TB treatment regimens, TB treatment outcomes, and HIV-related information (infection status, CD4, use of OI prophylaxis or ART). Data were analyzed using the Statistical Package for Social Sciences (SPSS) for Windows, version 12 software (SPSS Inc., Chicago, IL, USA) for analysis. Rates of childhood TB (per 100,000) were determined from surveillance data and population estimates; when calculating rates, we excluded reports from the national infectious diseases hospital, which has no population denominator. We classified age as 0–4 and 5–14 consistent with international recommendations [13]. Differences between groups were compared with X^2 ^test for categorical variables within different age groupings; where appropriate, Fisher's exact test was used. Statistical significance was defined as p < 0.05. Factors significant in univariate analysis or factors likely to be associated with outcomes were included in multivariate models using logistic regression, and automated backward, stepwise logistic regression was used to construct final models. We calculated odds ratios (OR), adjusted OR (AOR) and 95% confidence intervals (CI).

### Ethical review

The protocol for this demonstration project was reviewed by the Thailand MOPH and CDC and found to be surveillance and public health program implementation not requiring oversight by a human subjects research institutional review board.

## Results

### Case finding and incidence

From October 2004 to September 2006, 14,487 TB cases were recorded in surveillance. Of these, 279 (2%) cases occurred in children, including: 56 (20%) pulmonary, smear-positive; 103 (37%) pulmonary, smear-negative; 38 (14%) pulmonary, smear not known or done; and 82 (29%) extra-pulmonary. Lymph nodes were the most common extra-pulmonary site (Table 1). Most (78%) were registered as new cases. From October 2004 to September 2005, the incidence per 100,000 for all new TB cases was 14 for children, 12 for age 0–4, and 14 for age 5–14. For pulmonary TB, the incidence per 100,000 was 10 (3 smear-positive) for all children, 8 (1 smear-positive) for age 0–4, and 10 (3 smear-positive) for age 5–14. For extra-pulmonary TB, the incidence per 100,000 was 5 for age 0–4, and 4 for age 5–14.

**Table 1:** Characteristics of 279 childhood TB patients reported in the Thailand TB active surveillance network, October 2004 to September 2006

**Characteristic**	**Patients **No. (%)
Type, anatomic site of TB	
Smear-positive, pulmonary	56 (20)
Smear-negative, pulmonary	103 (37)
Smear-unknown, pulmonary	38 (14)
Extra-pulmonary	82 (29)
Disseminated	1 (0)
Pleural	2 (1)
Lymphatic	46 (16)
Bone/Joint	4 (1)
Meningeal	22 (8)
Peritoneal	1 (0)
Other/missing	6 (4)
Category of TB	
New	217 (78)
Relapse	3 (1)
Failure	1 (0)
Treatment after default	3 (1)
Transfer in	9 (3)
Other	46 (17)
Median age (years) (range)	8 (4 months-14 years)
Age group (years)	
< 1	9 (3)
1–4	65 (23)
5–9	87 (31)
10–14	118 (42)
Male	144 (52)
Non-Thai nationality	36 (13)
Mobile within the past 6 months	64 (23)
Median number of persons living in home, persons (range)	4 (1–170)
Median number of additional children < 5 years old living in home, persons (range)	0 (0–10)
Previously treated for TB	17 (6)
Previously treated with isoniazid for latent TB infection	12 (4)
Cough lasting > 2 weeks at time of diagnosis	121 (43)
Living in migrant or refugee camp	1 (0)
Facility that made TB diagnosis	
Private hospital	6 (2)
Government hospital or clinic	272 (98)
Unknown	1 (0)
Facility that provided TB treatment	
Private hospital	5 (2)
Government hospital or clinic	273 (98)
Unknown	1 (0)
Chest radiograph	
Normal	16 (6)
Not performed	31 (11)
Unknown	40 (14)
Abnormal	192 (69)
Presence of a cavity	16 (6)
Microbiological characteristics of pulmonary TB cases	
Smear-positive and culture-positive	34 (17)
Smear-positive and culture-negative or culture not done	22 (11)
Smear-negative and culture-positive	7 (4)
Neither smear- nor culture-positive	134 (68)
Initial treatment prescribed	
CAT I (2HRZE/4HR)	164 (59)
CAT II (2HRZES/1HRZ/4HR)	8 (3)
CAT III (2HRZ/4HR)	84 (30)
CAT IV (2HRZS/4HR)	8 (3)
Other/missing	15 (5)
Observer assigned during first 2 months of treatment	
Health care or village health worker	39 (14)
Family	220 (79)
None	17 (6)
Other/missing	3 (1)
Offered HIV counseling after diagnosis of TB	
Yes	148 (53)
No	46 (17)
HIV status already known	68 (24)
Unknown/missing	17 (6)
HIV status	
Infected	75 (27)
Uninfected	102 (37)
Unknown	102 (37)
Among pulmonary smear-positive patients, sputum converted to smear-negative after 2 months of treatment	36 (64)
Treatment outcome	
Success (completed, cured)	200 (72)
Failure	3 (1)
Died	17 (6)
Default	39 (14)
Transferred out	20 (7)

Of 74 children aged 0–4, 8 (11%) had pulmonary, smear-positive TB, compared with 48 (23%) of 205 children aged 5–14 (p < 0.01). The proportion with EPTB and sites involved did not differ by age group.

### Characteristics of cases

The median age was 8 years, and only 9 (3%) were younger than 1 year (Table 1). Half were male. Non-Thais, e.g., migrants, refugees, visitors, or other foreigners, accounted for 13% of cases. Only 2% of cases were diagnosed at a private facility. Previous treatment for TB disease was documented in 17 (6%) and for latent TB infection in 12 (4%). Almost half (43%) had cough lasting more than 2 weeks, and most (69%) had abnormal chest radiographs.

Of 197 pulmonary TB cases, 159 (81%) had at least one sputum smear performed, and 79 (50%) of these had at least one sputum culture performed. Of cases with a culture performed, 41 (52%) were culture-positive for *Mycobacterium tuberculosis*. Overall, 34 (17%) of the pulmonary TB cases were smear-positive and culture-positive, 22 (11%) were only smear-positive (4 culture-negative; 5 culture not done; 4 culture-positive for non-tuberculous mycobacterium [NTM]; and 9 culture unknown/missing), 134 (68%) were neither smear nor culture positive (24 culture-negative; 38 culture not done; 15 neither smear nor culture done; 57 culture unknown/missing), and 7 (4%) were smear-negative and culture-positive. Of 41 culture-positive cases, 33 (80%) had DST performed. MDR TB was diagnosed in 1, which was 0.5% of all 197 pulmonary TB cases and 3% of the 33 culture-positive cases that had DST performed.

### TB/HIV

Of 279 TB cases, 68 (24%) had known HIV status before their diagnosis of TB. Of the remaining 211 cases with unknown HIV status, 148 (53%) underwent HIV counseling; 108 (73%) of those counseled agreed to HIV testing. In all, 75 (27%) cases were known to be HIV-infected (55 or 73% of whom knew their HIV status before TB diagnosis), 102 (37%) were known to be HIV-uninfected, and 102 (37%) had unknown HIV status.

Of the 75 HIV-infected TB patients, 21 (28%) were pulmonary, smear-positive; 32 (43%) smear-negative; 7 (9%) smear not known or done and 15 (20%) extra-pulmonary. Most (77%) were new cases. Half were male, and 81% were more than 5 years old. CD4 counts were available for 46 (61%) cases. The median was 55 (range: 2; 2731) and mean 193 cells/mm^3^. The distribution of CD4 was similar in those 0–4 years old (in whom absolute CD4 is known to be unreliable) compared with those 5–14 years old. Only 6 (16%) of children aged 5–14 years had CD4 greater than 200 cells/mm^3^. ART was prescribed to 17 (23%) before the diagnosis of TB and to 16 (21%) during TB treatment. ART was not prescribed to 18 (24%) and was not known for 24 (32%). Of 32 HIV-infected children aged 5–14 years who had CD4 less than 200 cells/mm^3^, 20 (62%) received ART. Among children receiving ART, about half (45%) received a combination regimen of stavudine, lamivudine, and nevirapine. Rates of co-trimoxozole use were higher than ART; 23 (31%) were taking co-trimoxazole before their diagnosis of TB, and 21 (28%) were prescribed co-trimoxazole during TB treatment. Almost all (97%) children with CD4 less than 200 cell/mm^3 ^received co-trimoxazole.

### Treatment outcomes

Of all 279 patients, 200 (72%) were cured or completed TB treatment, 17 (6%) died during TB treatment, 3 (1%) failed treatment, 39 (14%) defaulted, and 20 (7%) transferred out (Table 1). We stratified outcomes by age group and HIV status (Table 2). More defaults occurred in children aged 0–4 (16/74, 22%) than in children aged 5–14 (23/205, 11%), (p < 0.02). No deaths occurred in the 74 children aged 0–4, compared with 17 (8%) deaths in the 205 children aged 5–14 (p = 0.03). Of the 17 deaths, 13 were in patients with pulmonary TB (2 culture-positive and smear-negative, 5 smear-positive only, 6 neither smear- nor culture-positive) and 4 were in extra-pulmonary TB (2 lymph node, 2 not known). Thirteen (76%) deaths occurred in HIV-infected children; of the nine with CD4 recorded, all had CD4 < 65 cells/mm^3^.

**Table 2:** Treatment outcomes for childhood TB cases, stratified by age group and HIV status, in Thailand TB active surveillance network, October 2004 to September 2006

**Age (years)**	**HIV status**	**Total**	**Treatment outcome No. (%) registered**				
			
			**Cured or completed**	**Failed**	**Died**	**Defaulted**	**Transferred out**
0–4	Positive	14	12 (86)	0 (0)	0 (0)	2 (14)	0 (0)
	Negative	30	16 (53)	0 (0)	0 (0)	9 (30)	5 (17)
	Unknown	30	23 (76)	0 (0)	0 (0)	5 (17)	2 (7)

5–14	Positive	61	37 (61)	0 (0)	13 (21)	7 (11)	4 (7)
	Negative	72	52 (72)	3 (4)	2 (3)	10 (14)	5 (7)
	Unknown	72	60 (83)	0 (0)	2 (3)	6 (8)	4 (6)

Total		279	200 (72)	3 (1)	17 (6)	39 (14)	20 (7)

We also stratified treatment outcomes by anatomic site and/or microbiologic characteristics (Table 3). In children with extra-pulmonary TB, treatment success rates were highest in patients with lymph node TB (86%). Of patients with meningitis, 67% had unknown outcomes (6 defaulted; 7 transferred out). All 13 patients with unknown outcome were from one province that borders Burma and has a large population of migrant Thais and non-Thais; 8 (62%) of these were non-Thai migrants. In children with pulmonary TB, treatment success rates were not significantly different when we stratified cases by microbiologic characteristics.

**Table 3:** Treatment outcomes for childhood TB cases, categorized by anatomic site and/or microbiologic characteristics, in Thailand TB active surveillance network, October 2004 to September 2006

**Diagnosis**	**Total**	**Treatment outcome No. (%) registered**				
		
		**Cured or completed**	**Failed**	**Died**	**Defaulted**	**Transferred out**
All types of TB	279	200 (72)	3 (1)	17 (6)	39 (14)	20 (7)
Pulmonary	197	144 (73)	3 (1)	13 (7)	26 (13)	11 (6)
Neither smear nor culture Positive	134	99 (74)	1 (1)	6 (4)	19 (14)	9 (7)
Culture or smear positive	41	32 (78)	1 (2)	2 (5)	4 (10)	2 (5)
Smear positive only	22	13 (59)	1 (4)	5 (23)	3 (14)	0 (0)
Lymph node	42	36 (86)	0	2 (5)	4 (9)	0
Meningitis	21	8 (38)	0	0	6 (29)	7 (33)
Other types of ETB	19	12 (63)	0	2 (10)	3 (16)	2 (10)

### Factors associated with death or default

In univariate and multivariate analysis comparing patients who died with those successfully treated, factors statistically associated with death were treatment at the national infectious diseases hospital (aOR 7.3; CI, 1.3–41.6, compared with treatment at other sites), and being HIV-infected (aOR 6.9; CI, 1.4–33.0, compared with HIV-uninfected). No children aged less than 5 years died compared with 17% of children aged 5 – 14 years (Table 4). In univariate and multivariate analysis comparing patients who defaulted with those successfully treated, factors statistically associated with default were being non-Thai (aOR 5.8; CI, 2.3–14.8) compared with being Thai (Table 5).

**Table 4:** Factors associated with death versus successful (cure or completed) outcome among childhood TB cases in Thailand, October 2004 to September 2006

**Characteristic**	**No. died/total (%)**	**Unadjusted OR (95% CI)**	**Adjusted OR (95% OI)**
Age (in years)			
< 5	0/51 (0)	-	-
5–14	17/166 (10)	-	
Gender			
Male	12/111 (11)	2.8 (0.8–9.7)	-
Female	5/106 (5)	Referent	
Nationality			
Non-Thai	1/15 (7)	1.0 (0.1–11.2)	-
Thai	16/202 (8)	Referent	
Site			
National infectious diseases hospital	4/8 (50)	7.4 (0.9–58.7)	7.3 (1.3–41.6)
Chiang Rai	6/104 (6)	0.8 (0.2–2.9)	0.8 (0.3–2.6)
Other	7/105 (7)	Referent	Referent
Diagnosis			
Pulmonary, smear-positive	6/45 (13)	1.5 (0.4–6.0)	-
Pulmonary, smear-negative	7/80 (9)	Referent	
Pulmonary, smear-unknown	0/32 (0)	-	
Extra-pulmonary TB	4/60 (7)	0.7 (0.1–3.4)	
Registration status			
New	15/167 (9)	1.6 (0.2–10.7)	-
Previously treated	2/50 (4)	Referent	
HIV status			
Positive	13/62 (21)	5.9 (1.2–30.1)	6.9 (1.4–33.0)
Negative	2/70 (3)	Referent	Referent
Unknown	2/85 (2)	0.9 (0.1–7.4)	0.7 (0.1–5.4)

**Table 5:** Factors associated with default versus successful (cure or completed) outcome among childhood TB cases in Thailand, October 2004 to September 2006

**Characteristic**	**No. defaulted/total (%)**	**Unadjusted OR (95% CI)**	**Adjusted OR (95% OI)**
Age (in years)			
< 5	16/67 (24)	2.0 (0.9–4.5)	2.0 (0.9–4.5)
5–14	23/172 (13)	Referent	Referent
Gender			
Male	23/122 (19)	1.5 (0.7–3.3)	-
Female	16/117 (14)	Referent	
Nationality			
Non-Thai	13/27 (48)	6.3 (2.3–16.8)	5.8 (2.3–14.8)
Thai	26/212 (12)	Referent	Referent
Site			
National infectious diseases hospital	1/5 (20)	0.6 (0.0–9.2)	0.6 (0.0–8.7)
Chiang Rai	25/123 (20)	0.5 (0.1–1.5)	0.5 (0.2–1.6)
Phuket	1/25 (4)	0.1 (0.0–1.0)	0.1 (0.0–1.1)
Ubon-ratchathani	5/58 (9)	0.3 (0.1–1.3)	0.3 (0.1–1.4)
Bangkok	7/28 (25)	Referent	Referent
Diagnosis			
Pulmonary, smear-positive	7/46 (15)	0.5 (0.2–1.6)	-
Pulmonary, smear-negative	14/87 (16)	Referent	
Pulmonary, smear-unknown	5/37 (13)	1.9 (0.3–11.2)	
Extra-pulmonary TB	13/69 (19)	1.0 (0.4–2.7)	
Registration status			
New	32/184 (17)	2.1 (0.5–8.6)	-
Previously treated	7/55 (13)	Referent	
HIV status			
Positive	9/58 (15)	0.8 (0.3–2.1)	0.8 (0.3–2.1)
Negative	19/87 (22)	Referent	Referent
Unknown	11/94 (12)	0.3 (0.1–0.8)	0.4 (0.1–1.0)

## Discussion

We found that childhood TB is infrequently diagnosed in Thailand. In our population, the rate of childhood TB was 14 cases per 100,000, consistent with one published estimate, derived from the WHO global report, of childhood TB incidence in Thailand (15 cases per 100,000 children) [3]. The rate of smear-positive TB in our population was much higher (3 per 100,000 versus 1 per 100,000) than routine national surveillance data. There are at least two possible explanations for the higher case detection rate in our project compared with national surveillance. First, national surveillance may not sufficiently capture all diagnosed cases of TB. Our demonstration project involved contacting healthcare facilities regularly to ascertain diagnosed cases of TB; it is likely that we identified cases of TB that would not normally have been reported [14]. Second, and conversely, our surveillance sites may over-estimate the overall burden of childhood TB in Thailand, because the participating provinces have higher TB and HIV prevalence rates than the national average and have enhanced TB laboratory capacity. Enhancing the national surveillance system to collect age data for all forms of TB, to differentiate between children aged 0–4 and 5–14, to collect microbiological characteristics of pulmonary TB cases, and to integrate HIV-related data would permit more detailed analysis and better define the burden of childhood TB in Thailand. We found a low rate of extra-pulmonary TB compared to other reports [4]. It is possible that high coverage of BCG vaccination can prevent invasive TB disease. We did not collect data on history of BCG vaccination or BCG scar; however, national coverage of BCG vaccine in 2006 was more than 99% [15].

Unfortunately, measuring the true burden of childhood TB in any country is extremely difficult, because no diagnostic test performs well in childhood TB. Most children cannot expectorate sputum and/or have pauci-bacillary pulmonary TB. Gastric aspiration and sputum induction, although validated, are used mainly in academic or big referral hospitals and are infrequently used in small hospitals because of technical demands and relatively low yield [16]. In our project, most cases were not bacteriologically-confirmed, consistent with data from both low- and high-burden TB countries [6]. International guidelines also instruct clinicians to use radiographic changes, tuberculin skin testing, symptoms, and contact with a known TB case as clues to the diagnosis [7]. Our enhanced surveillance system did not record sufficient detail about these characteristics and gastric aspiration to allow us to assess the accuracy of TB diagnosis. Further, because contact investigations are rarely performed in Thailand, many childhood TB cases may have gone undiagnosed in the community. A more detailed evaluation of clinical diagnostic and public health practices in Thailand is needed to understand the accuracy of childhood TB diagnosis and the relative contribution of diagnostic effort to differences in reporting rates.

HIV contributes substantially to the burden of childhood TB in Thailand. The minimum prevalence of HIV infection in children (27%) was similar to that in adults (31%) recorded in our surveillance system (Thailand MOPH, unpublished data). The HIV prevalence was higher in older children than in younger children, likely because of the effectiveness of Thailand's highly effective mother-to-child HIV transmission that began in 2000 [17]. Almost one-fifth of HIV-infected children with TB died during TB treatment, all of them aged 5–14. The markedly advanced immune-suppression in these children is identical to that seen in HIV-infected Thai adults, in whom TB occurs almost exclusively at low CD4 levels [14,18]. Our data shows that despite national TB and HIV treatment guidelines, coverage of some key aspects of TB and HIV care was low. As in adults, reducing TB morbidity and mortality in HIV-infected children requires further integration of HIV counseling and testing into TB services, ART and co-trimoxazole for HIV-infected TB patients, prevention of TB through treatment of latent TB infection, and earlier diagnosis through contact investigations and routine screening for TB disease in HIV-infected children. Documenting CD4 percentage, as well as absolute count, on TB registers will assist in identifying when children aged less than 5 years should be receiving ART as well as TB treatment.

Data on childhood TB treatment outcomes is scarce. One surprising finding was the high rate of default particularly patients with meningitis. Most defaults occurred in non-Thais, a heterogeneous group of 2.3 million people that consist of economic migrants, refugees, visitors, and other temporary residents [19]. Because rates of default are known to be high in adult migrants with TB, our finding is worrisome, albeit expected. Further efforts are needed to maintain non-Thai adults and children on TB treatment to prevent drug resistance.

Our study has important limitations, particularly its small sample size and the absence of microbiologic confirmation in most diagnosed patients. Its primary strength is that it is the only population-based study that we know of about childhood TB epidemiology and treatment outcomes in Asia, a region that contributes to one-half of the global TB burden. Our data confirm that the burden of diagnosed childhood TB in Thailand is less than that in other high-burden countries, but it is substantially greater than national surveillance data indicate and is closely associated with the HIV epidemic. Strengthening national surveillance to capture all diagnosed cases of TB in children, providing training or guidance to health care providers in community hospitals how to perform gastric aspiration and to record more detailed data about childhood TB cases will help provide more accurate estimates of the national disease burden and potentially increase political commitment to address childhood TB. Reducing morbidity and mortality from TB in Thai children will require enhancing laboratory capacity for microbiologic confirmation of disease, increasing TB screening in household contacts and HIV-infected children, strengthening linkages to HIV-related services, and reducing default rates.

## Conclusion

Childhood TB is infrequently diagnosed in Thailand. Understanding whether this is due to absence of disease or diagnostic effort requires further research. Strengthening national surveillance to capture all diagnosed cases of TB in children will help provide more accurate estimates of the national disease burden and potentially increase political commitment to address childhood TB. HIV contributes substantially to the childhood TB burden in Thailand and is associated with high mortality. Reducing morbidity and mortality from TB in Thai children will require enhancing laboratory capacity for microbiologic confirmation of disease, increasing TB screening in household contacts, reducing default rates and strengthening linkages to HIV-related services for HIV-infected children.

## Competing interests

The authors declare that they have no competing interests. Funding for this project was from the Thailand Ministry of Public Health, the U.S. Centers for Disease Control and Prevention, and the Research Institute of Tuberculosis (Japan). The authors of this manuscript are members of those public health agencies. Additional funding support was from the U.S. Agency for International Development, which played no role in design, analysis, or manuscript preparation for this study. The findings and conclusions in this manuscript are those of the authors and do not necessarily represent the views of the Centers for Disease Control and Prevention.

## Authors' contributions

RL performed statistical analysis, interpretation of data, and drafting and revision of the manuscript. AA participated in study design, statistical analysis, and data collection. SN, SS, NY, WW, WS, PC, NS participated in study design and data collection. CDW participated in study design and revision of the manuscript. JKV participated in study design, interpretation of data, and drafting and revision of the manuscript. All authors approved the final version of the manuscript.

## Pre-publication history

The pre-publication history for this paper can be accessed here:


